# Plasma oligomer beta-amyloid is associated with disease severity and cerebral amyloid deposition in Alzheimer’s disease spectrum

**DOI:** 10.1186/s13195-024-01400-3

**Published:** 2024-03-11

**Authors:** Sheng-Min Wang, Dong Woo Kang, Yoo Hyun Um, Sunghwan Kim, Chang Uk Lee, Philip Scheltens, Hyun Kook Lim

**Affiliations:** 1 Department of Psychiatry, Yeouido St. Mary’s Hospital, College of Medicine, The Catholic University of Korea, 10, 63-Ro, Yeongdeungpo-Gu, Seoul, 07345 South Korea; 2 Department of Psychiatry, Seoul St. Mary’s Hospital, College of Medicine, The Catholic University of Korea, Seoul, 06591 South Korea; 3 Department of Psychiatry, St. Vincent Hospital, Suwon, Korea, College of Medicine, The Catholic University of Korea, Suwon, 16247 South Korea; 4 Alzheimer Center Amsterdam, Neurology, Vrije Universiteit Amsterdam, Amsterdam UMC location VUmc, Boelelaan 1118, Amsterdam, 1081, HZ Netherlands; 5 EQT Life Sciences Partners, Amsterdam, 1071, DV The Netherlands

**Keywords:** Oligomerization, Blood-based biomarker, Beta amyloid, Mild cognitive impairment, Dementia, And Alzheimer’s disease

## Abstract

**Background:**

Multimer detection system-oligomeric amyloid-β (MDS-OAβ) is a measure of plasma OAβ, which is associated with Alzheimer’s disease (AD) pathology. However, the relationship between MDS-OAβ and disease severity of AD is not clear. We aimed to investigate MDS-OAβ levels in different stages of AD and analyze the association between MDS-OAβ and cerebral Aβ deposition, cognitive function, and cortical thickness in subjects within the AD continuum.

**Methods:**

In this cross-sectional study, we analyzed a total 126 participants who underwent plasma MDS-OAβ, structural magnetic resonance image of brain, and neurocognitive measures using Korean version of the Consortium to Establish a Registry for Alzheimer’s Disease, and cerebral Aβ deposition or amyloid positron emission tomography (A-PET) assessed by [^18^F] flutemetamol PET. Subjects were divided into 4 groups: *N* = 39 for normal control (NC), *N* = 31 for A-PET-negative mild cognitive impairment (MCI) patients, *N* = 30 for A-PET-positive MCI patients, and *N* = 22 for AD dementia patients. The severity of cerebral Aβ deposition was expressed as standard uptake value ratio (SUVR).

**Results:**

Compared to the NC (0.803 ± 0.27), MDS-OAβ level was higher in the A-PET-negative MCI group (0.946 ± 0.137) and highest in the A-PET-positive MCI group (1.07 ± 0.17). MDS-OAβ level in the AD dementia group was higher than in the NC, but it fell to that of the A-PET-negative MCI group level (0.958 ± 0.103). There were negative associations between MDS-OAβ and cognitive function and both global and regional cerebral Aβ deposition (SUVR). Cortical thickness of the left fusiform gyrus showed a negative association with MDS-OAβ when we excluded the AD dementia group.

**Conclusions:**

These findings suggest that MDS-OAβ is not only associated with neurocognitive staging, but also with cerebral Aβ burden in patients along the AD continuum.

## Background

Alzheimer’s disease (AD) is the most common cause of dementia, accounting for 60 to 80 percent of all cases [1]. The classic pathophysiological hallmarks of AD, including β-amyloid (Aβ), tau protein, and neurodegeneration, can be measured using cerebrospinal fluid (CSF) studies and imaging techniques [2]. With the recent approval of disease-modifying drugs targeting Aβ in the brain, such as aducanumab in 2021 [3] and lecanemab in 2023 [4], biomarker-based studies are receiving increased attention. Moreover, serial measurements of biomarkers are needed for drug development trials and applicability to real clinical settings. However, PET imaging of Aβ, tau and fluorodeoxyglucose (FDG), and CSF studies of β-amyloid 42 (Aβ_42_) and 40 (Aβ_40_), phosphorylated tau (p-Tau), and total tau (t-Tau) are expensive, invasive, or both, which hinders repetitive collections of these biomarkers. Thus, many studies have increasingly focused on blood-based biomarkers, with recent work showing promising results of showing correlation with cerebral Aβ deposition by measuring blood level of Aβ [5] and p-Tau (pT181, pT217, and pT231) [6–8].

Multimer detection system-oligomeric Aβ (MDS-OAβ) can measure the oligomerization dynamics or oligomerization tendencies in plasma samples after spiking synthetic Aβ [9]. MDS-OAβ selectively detects oligomeric forms of Aβ (OAβ) [10, 11]. Research has repeatedly shown that patients with dementia due to AD, also called AD dementia, had a higher plasma concentration of MDS-OAβ compared to normal control (NC) [12, 13]. The study also found a positive correlation between MDS-OAβ and cerebral Aβ deposition severity measured using the standardized uptake value ratio (SUVR) of Pittsburgh compound B (PiB) (MDS-OAβ with PiB SUVR; *r* = 0.430) [12]. A more recent voxel-based morphometry (VBM) study further showed that MDS-OAβ level had a correlation with brain volume reduction in cortical regions of AD [14]. However, the relationship between MDS-OAβ level and disease severity along the AD continuum is not clear. A study showed that MDS-OAβ had a negative correlation with cognitive function, but MDS-OAβ did not differ between NC and mild cognitive impairment (MCI) patients [15]. In contrast, others found that MCI and dementia patients with positive amyloid positron emission tomography (A-PET) had higher MDS-OAB level than NC, but MDS-OAβ level showed a decreasing trend as the clinical dementia rating (CDR) score increased from 0.5 to 1 and 2 [16].

A possible explanation for these contradictory results is that subjects’ disease severities were not precisely stratified. One of the two studies did not utilize biomarkers, such as A-PET or CSF Aβ measures, and grouped the subjects merely based on neurocognitive measures [15]. Inevitably, a significant proportion of subjects in the NC and MCI groups might have shown overt cerebral Aβ deposition if they were tested with A-PET or CSF Aβ studies. The second study included subjects who underwent A-PET, but all MCI subjects included were A-PET-positive [16]. Thus, the study failed to elucidate whether the difference in MDS-OAβ was attributed to the cerebral Aβ burden, neurocognitive function, or mixture of the two. Moreover, none of the previous studies compared MDS-OAβ between A-PET-positive MCI and A-PET-negative MCI. Likewise, the cerebral Aβ burden of the subjects in the VBM analysis was also not confirmed using either A-PET or CSF studies. Moreover, only 3% (14/162) of subjects had a diagnosis of MCI [14]. Due the skewness of subjects, the VBM study was not able to correctly investigate association between the MDS-OAβ level with that of cortical atrophy in the AD continuum. The fact that VBM is known to be more affected by diverse cortical gray matter pathologies when compared to the cortical thickness measurement is another important shortcoming [17].

To fill in this gap, we investigated whether MDS-OAβ differed according to stage of AD. We hypothesized that MDS-OAβ level would be higher in patients with significant cognitive impairment (MCI to dementia), and MDS-OAβ would differ according to cerebral Aβ in MCI. We also investigated the association between MDS-OAβ and cerebral Aβ deposition, neurocognitive measures, and cortical thickness in subjects along the AD continuum.

## Methods

### Subjects

A total of 122 subjects consisting of 39 A-PET-negative cognitively normal older adults (normal control: NC), 31 A-PET-negative MCI patients, 30 A-PET-positive MCI patients, and 20 A-PET-positive dementia patients (AD dementia) were included in the study. Subjects were recruited from volunteers in the Catholic Aging Brain Imaging (CABI) database, which contains the brain scans of patients who visited the outpatient clinic at Catholic Brain Health Center, Yeouido St. Mary’s Hospital, The Catholic University of Korea, between 2017 and 2022. The inclusion criteria for all subjects were as follows: [1] age ≥ 55 years and [2] no clinically significant psychiatric disorders (depressive disorder, schizophrenia, or bipolar disorder). In terms of NCs, they visited our outpatient clinic to undergo a brain examination as part of the health checkup. Their normal cognitive functions were confirmed with the Korean version of the Consortium to Establish a Registry for Alzheimer’s Disease (CERAD-K), which includes a verbal fluency (VF) test, the 15-item Boston Naming Test (BNT), the Korean version of the Mini-Mental State Examination (MMSE), word list memory (WLM), word list recall (WLR), word list recognition (WLRc), constructional praxis (CP), and constructional recall (CR) [18]. The criteria for MCI were as follows: (1) presence of memory complaints corroborated by an informant; (2) objective cognitive impairment in more than one cognitive domain on CERAD-K (at least 1.0 standard deviation (SD) below age- and education-adjusted norms), (3) intact activities of daily living (ADL); (4) CDR of 0.5; and (5) not demented according to the Diagnostic and Statistical Manual of Mental Disorders (DSM)-V criteria. The patients with AD dementia met the probable AD criteria proposed by the National Institute of Neurological and Communicative Disorders and Stroke and AD and Related Disorders Association (NINCDS- ADRDA) [19], as well as those proposed by the DSM-V with A-PET-positive results [20].

We excluded subjects with the following: (1) systemic diseases that can cause cognitive impairment, such as thyroid dysfunction, severe anemia, and syphilis infection; (2) severe hearing or visual impairment; (3) other neurological diseases that can cause cognitive impairment, such as brain tumor, encephalitis, and epilepsy; (4) clinically significant cerebral infarction or cerebral vascular disease; (5) prescription medications that may cause changes in cognitive function; and (6) contraindications for magnetic resonance imaging (MRI) examination. Diagnoses of cognitively normal status, MCI, and dementia were conducted separately by two psychiatric specialists, and they also confirmed the inclusion and exclusion criteria. The study was conducted in accordance with the ethical and safety guidelines set forth by the Institutional Review Board of Yeouido St. Mary’s Hospital, The Catholic University of Korea (IRB number: SC21TISI0017), and all subjects provided written informed consent.

### Measurement of Aβ oligomerization in plasma

MDS-OAβ was used to measure the plasma level of OAβ. We used an ethylene-diamine-tetraacetic acid (EDTA) vacutainer tube to collect subjects’ blood plasma through venipuncture. In terms of the sampling process, we followed a previous procedure that used EDTA to measure MDS-OAβ level [21]. The EDTA plasma was centrifuged at 3500 rotations per minute for 15 min at room temperature, and then was stored in 1.5-ml polypropylene tubes at a temperature between − 70 and − 80 °C. The samples were then sent to PeopleBio Inc. to assess the levels of MDS-OAβ. Before analysis, the plasma aliquots were defrosted for 15 min at 37 °C. The measurement of MDS-OAβs was performed utilizing the multimer detection system, which has received Conformité Européene (CE) marking and has been authorized by the Korean Food and Drug Administration [9–15].

### MRI acquisition and pre-processing for morphometric analysis

All study participants received MRI scans using a Siemens MAGETOM Skyra machine with Siemens head coils. The T1-weighted three-dimensional magnetization-prepared rapid gradient-echo (3D-MPRAGE) sequence used the following parameters: time to echo (TE) of 2.6 ms, repetition time (TR) of 1940 ms, inversion time of 979 ms, field-of-view (FOV) of 230 mm, matrix of 256 × 256, and voxel size of 1.0 × 1.0 × 1.0mm^3^. In terms of pre-processing, we utilized the FreeSurfer software (version 6.0.0, available online at https://surfer.nmr.mgh.harvard.edu) to perform cortical reconstruction and volumetric segmentation of the whole brain [22]. The process involved several steps, which have been described previously [23]. Briefly, the steps included removal of non-brain tissue using a hybrid watershed algorithm, bias field correction, automated Talairach transformation, and segmentation of subcortical white matter (WM) and deep gray matter (GM) structures. Afterward, we normalized the intensity and inflated the cortical surface of each hemisphere to locate both the pial surface and the GM/WM boundary, which allowed us to compute cortical thickness using the shortest distance between the two surfaces at each point across the cortical mantle [24]. For the entire cortex analyses, we smoothed the cortical map of each subject using a Gaussian kernel with a full width at half-maximum (FWHM) of 10 mm. Finally, we parcellated the cerebral cortex based on gyral and sulcal information implemented in FreeSurfer.

### Amyloid positron emission tomography

All participants received PET scans using ^18^F-flutemetamol (^18^F-FMM). Information about the production of ^18^F-FMM, data collection, and analytical results was previously described [25]. T1 MRI images were used for each participant to co-register, define regions of interest, and correct partial volume effects caused by cerebral atrophy. The analysis of ^18^F-FMM PET data was based on the standardized uptake value ratio (SUVR) 90 min post-injection. In terms of regional SUVR values, we measured six cortical regions of interest (frontal, superior parietal, lateral temporal, striatum, anterior cingulate cortex, and posterior cingulate cortex/precuneus) using PMOD Neuro Tool. Thereafter, the global Aβ burden in the brain was acquired by averaging the SUVR values of these six cortical ROIs using the PMOD Neuro Tool. Lastly, two nuclear medicine radiologists confirmed the presence of Aβ deposition using visual readings.

### Statistical analysis

We used a free and open-source data analysis tool, Jamovi (Version 2.3.18.0), to conduct statistical analysis [26]. We used the analysis of variance (ANOVA) to assess potential differences between groups (NC, A-PET-negative MCI, A-PET-positive MCI, and AD dementia) for continuous variables and the chi-square test for categorical variables. When the group difference was statistically significant, Bonferroni tests were utilized for post hoc analysis. A two-tailed *α* level of 0.05 was chosen to indicate statistical significance for all statistical tests.

## Results

### Baseline demographic and clinical data

Table 1 shows the baseline demographic data of the NC (*n* = 39), A-PET-negative MCI (*n* = 31), A-PET-positive MCI (*n* = 30), and AD dementia (*n* = 22) groups. All variables were normally distributed, and there were no significant differences in sex ratio and education level among the 4 groups. The NC group had a significantly lower age than the A-PET-positive MCI and AD dementia groups (*P* < 0.05 for ANOVA and for post hoc analysis with Bonferroni correction), but there were no significant differences in age among the A-PET-negative MCI, A-PET-positive MCI, and AD dementia groups. Global cerebral Aβ deposition, or global SUVR values, were significantly higher for A-PET-positive MCI and AD dementia groups than for CN and A-PET-negative MCI groups (*P* < 0.001 for ANOVA and *P* < 0.05 for post hoc analysis with Bonferroni correction). In terms of neuropsychological measures, group differences were noted in the order of CN > A-PET-negative MCI > A-PET-positive MCI > AD dementia groups (*P* < 0.001 for ANOVA and *P* < 0.05 post hoc analysis with Bonferroni correction).

**Table 1:** Demographic and clinical characteristics of the study participants

	**A-PET negative NC (*N* = 39)**	**A-PET negative MCI (*N* = 31)**	**A-PET positive MCI (*N* = 30)**	**AD dementia (*N* = 22)**	***P* value**
**Age (years ± SD)**	73.05 (5.68)	75.68 (6.41)	77.27 (7.22)	77.77 (6.93)	0.02
**Post hoc**^**a**^	NC < PET − MCI, PET + MCI, and AD dementia; others no statistical difference				
**Education (years ± SD)**	11.82 (4.56)	9.14 (4.71)	10.90 (5.40)	9.50 (6.07)	NS
**Sex (M:F)**	10:29	9:22	13:17	8:14	NS
**SUVR (mean ± SD)**	0.50 (0.076)	0.51 (0.069)	0.75 (0.081)	0.795 (0.094)	< .001
**Post hoc**^**a**^	NC = PET − MCI < PET + MCI and AD dementia				
** APOE4 (%)**	12.8	19.4	56.7	57.1	< .001
**CERAD-K Battery (SD)**					
VF	14.77 (4.05)	10.32 (4.23)	10.63 (2.74)	5.96 (3.15)	< .001
BNT	12.67 (1.72)	9.39 (3.32)	10.53 (2.33)	7.09 (3.96)	< .001
MMSE	28.23 (1.69)	23.36 (3.56)	22.27 (3.36)	16.14 (4.99)	< .001
WLM	18.69 (3.29)	13.94 (3.23)	13.37 (3.41)	8.77 (4.15)	< .001
CP	9.97 (1.29)	8.68 (1.81)	9.47 (1.74)	8.27 (2.33)	< .001
WLR	6.15 (1.44)	3.42 (1.65)	2.53 (1.87)	1.32 (1.43)	< .001
WLRc	9.23 (1.11)	7.23 (2.26)	6.13 (2.27)	3.32 (2.21)	< .001
CR	7.28 (2.32)	3.45 (2.38)	2.53 (3.25)	1.5 (2.24)	< .001
CERAD total score	71.49 (9.25)	52.97 (11.12)	52.67 (8.92)	34.73 (12.65)	< .001

^**a**^Bonferroni corrected for multiple corrections

*BNT* 15-Item Boston Naming Test, *CERAD-K* The Korean Version of Consortium to Establish A Registry For Alzheimer’s Disease, *CDR* Clinical Dementia Rating, *CP* Constructional Praxis, *CR* constructional recall, *MMSE* Mini-Mental Status Examination, *NS* not significant, *SD* standard deviation, *VF* verbal fluency, *WLRc* word list recognition, *WLM* word list memory, *WLR* word list recall

### Plasma MDS-OAβ, cerebral Aβ deposition, and neuropsychological measures

**Fig. 1:**
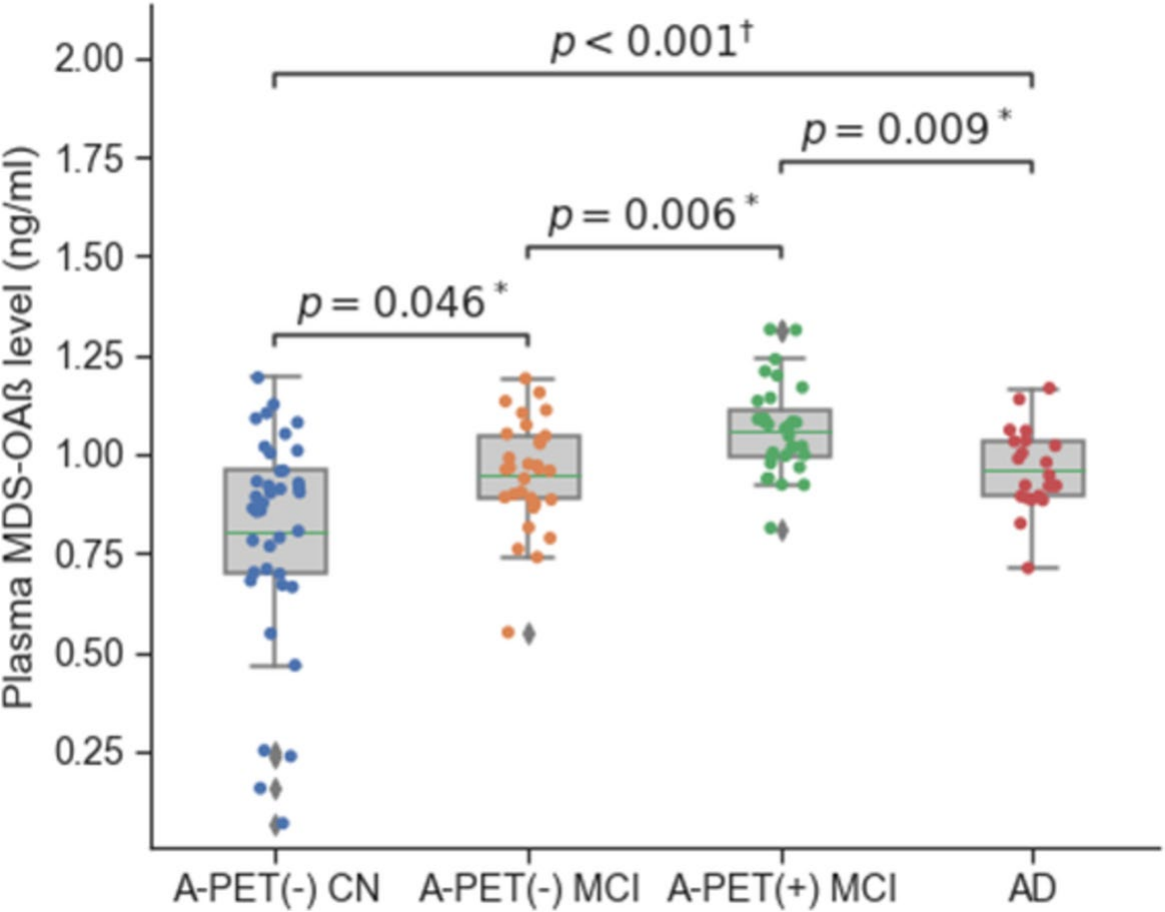
MDS-OAß level according to disease stage of AD ^+^ Analysis of variance (ANOVA), ^*^ Post hoc analysis with Bonferroni correction. AD: Alzheimer’s disease; A-PET: Amyloid-PET scan; CN: A-PET negative cognitive normal older adults; MCI: Mild cognitive impairment

There was a group difference in MDS-OAβ level (*P* < 0.001 for ANOVA). Post hoc analysis showed that MDS-OAβ level was highest in the A-PET-positive MCI group (1.07 ± 0.17) and lowest in the NC group (0.803 ± 0.27). MDS-OAβ level was not significantly different between A-PET-negative MCI (0.946 ± 0.137) and AD dementia (0.958 ± 0.103) groups (CN < A-PET-negative MCI & AD dementia < A-PET-positive MCI, for all *p* < 0.05 Bonferroni corrected (Fig. 1).

**Fig. 2:**
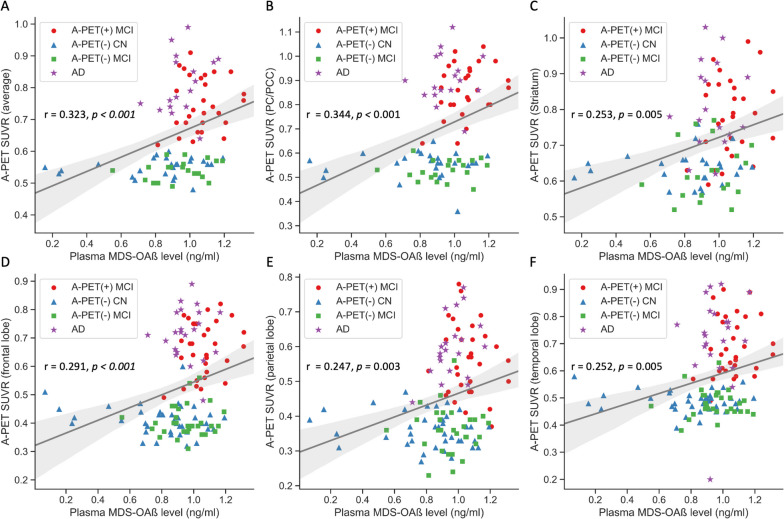
Association between MDS-OAß level and global and regional cerebral beta amyloid deposition in all subjects PC: Precuneus; PCC: Posterior cingulate cortex; SUVR: Standardized uptake volume ratio

**Fig. 3:**
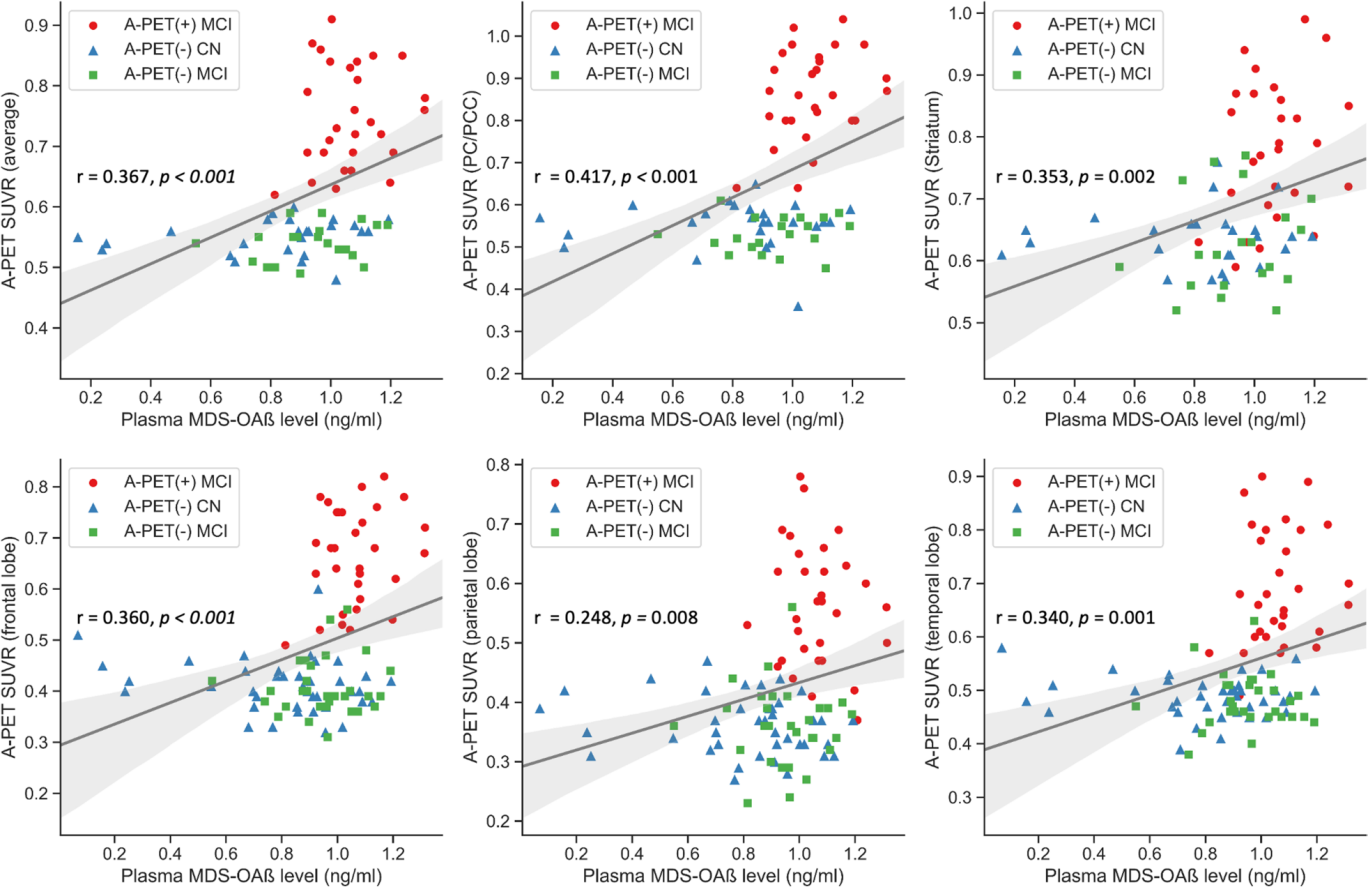
Association between MDS-OAß level and global and regional cerebral beta amyloid deposition in subjects with MCI only PC: Precuneus; PCC: Posterior cingulate cortex; SUVR: Standardized uptake volume ratio

Figure 2 shows the results of the correlation analysis between MDS-OAβ level and cerebral AB deposition level (SUVR). Global SUVR (*r* = 0.323, *p* < 0.001) and regional SUVR of the PCC/PC (*r* = 0.344, *p* < 0.001), striatum (r = 0.253, *p* < 0.01), frontal lobe (*r* = 0.291, *p* < 0.001), parietal lobe (r = 0.247, *p* < 0.01), and lateral temporal lobe (*r* = 0.252, *p* < 0.01) showed a positive correlation with MDS-OAβ (Fig. 2A ~ F). Since the group analysis showed that MDS-OAβ was lower in the AD dementia group than in the A-PET-positive MCI group, we conducted an additional correlation analysis after excluding patients with AD dementia. The positive association between MDS-OAβ and SUVR persisted with a higher correlation coefficient: Global SUVR (*r* = 0.367, *P* < 0.001) and regional SUVR of PCC/PC (*r* = 0.417, *p* < 0.001), striatum (*r* = 0.353, *p* = 0.002), frontal lobe (*r* = 0.360, *P* < 0.001), parietal lobe (*r* = 0.248, *p* = 0.008), and lateral temporal lobe (*r* = 0.340, *p* = 0.001) (Fig. 3).

**Fig. 4:**
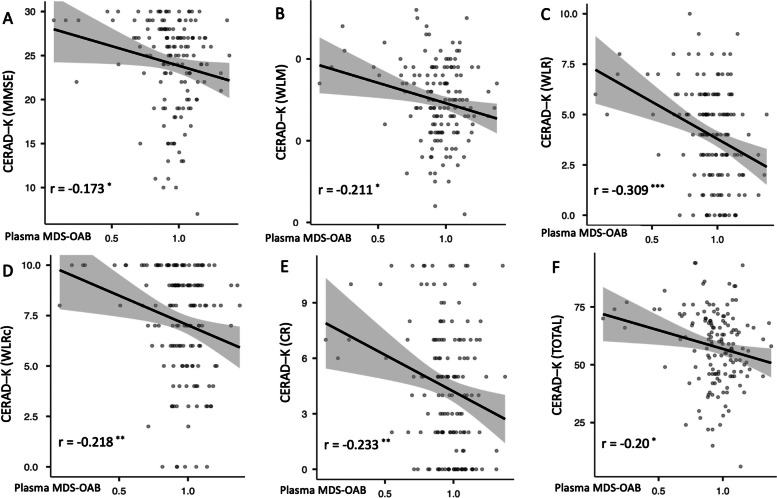
Association between MDS-OAß level and neuropsychological measure ^*^P < 0.05, ^**^P < 0.01, ^***^P < 0.001; CERAD-K: The Korean Version of Consortium to Establish A Registry For Alzheimer’s Disease; CR: Constructional Recall; MMSE: Mini-Mental Status Examination; WLRc: word list recognition; WLM: word list memory; WLR, word list recall

The associations between MDS-OAβ and neuropsychological measures of the CERAD-K were also analyzed (Fig. 4A ~ F). The results showed a negative correlation between MDS-OAβ and MMSE score (*r* =  − 0.173, *P* < 0.05), word list memory (*r* =  − 0.211, *P* < 0.05), word list recall (*r* =  − 0.309, *P* < 0.001), word list recognition (*r* =  − 0.218, *P* < 0.01), constructional recall (*r* =  − 0.233, *P* < 0.01), and total score (*r* =  − 0.20, *P* < 0.05).

### Cortical thickness and MDS-OAβ level

**Fig. 5:**
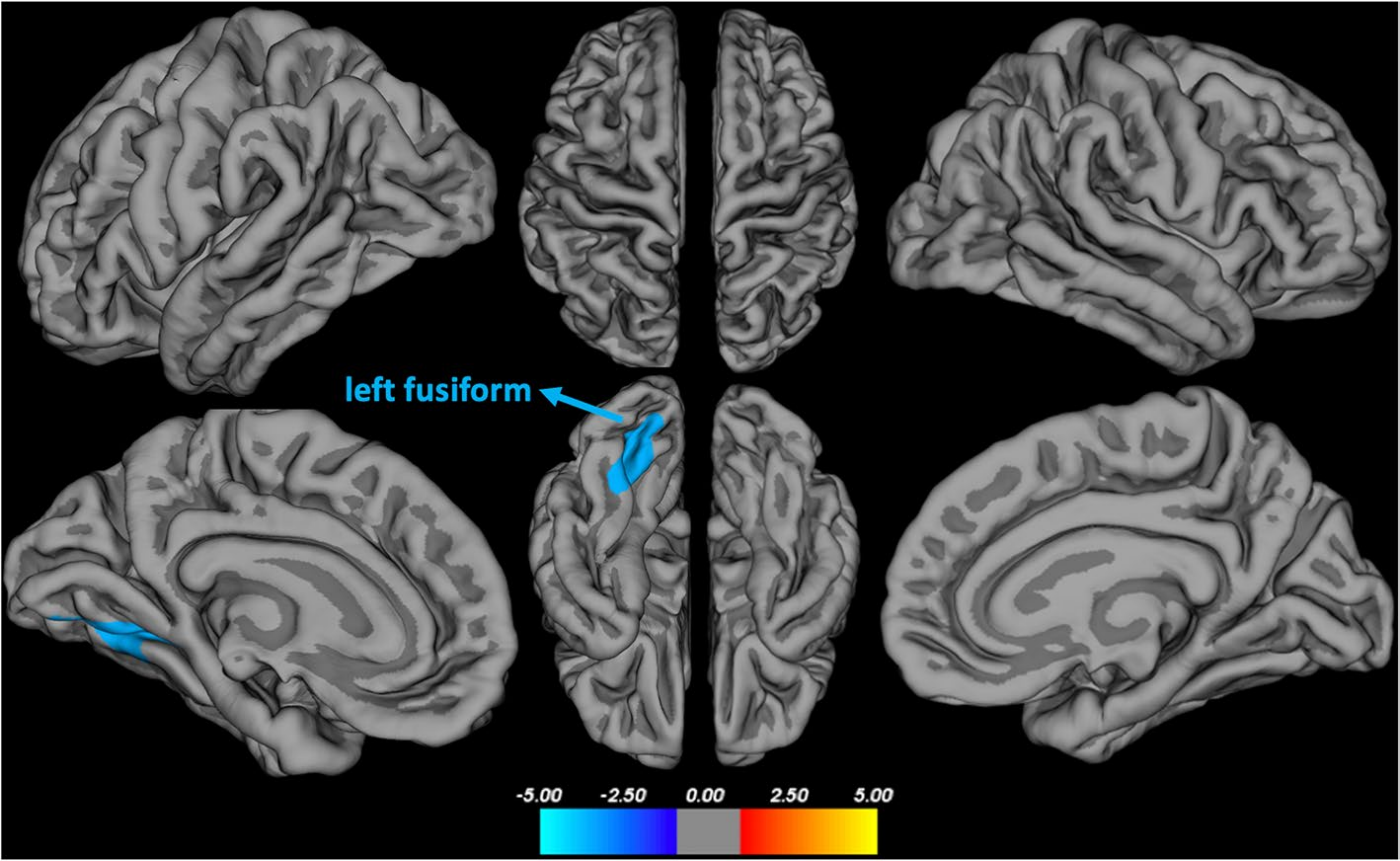
Association between MDS-OAß level and cortical thickness excluding AD dementia group Correlations analysis showed a negative correlation between plasma MDS-OAβ levels and left fusiform excluding AD dementia group (T-map, *p* < 0.05, thresholded at Monte Carlo Null-Z simulation)

There was no statistically significant association between MDS-OAβ and cortical thickness. When we excluded patients with AD dementia, MDS-OAβ showed a negative correlation with the cortical thickness of the left fusiform (age as a covariate; *p* < 0.05, multiple comparisons by Monte Carlo simulation; Fig. 5).

## Discussion

To the best of our knowledge, this is the first study to investigate MDS-OAβ in A-PET-confirmed patients having different stages of AD. In line with previous research showing that MDS-OAβ peaked in MCI and lowered as the disease progressed [16], we showed that MDS-OAβ was highest in the A-PET-positive MCI group. Previous studies have not distinguished patients’ cerebral Aβ status using A-PET or CSF studies. Thus, we are the first to show that MDS-OAβ was higher in subjects with A-PET-positive MCI (1.07 ± 0.17) than in subjects with A-PET-negative MCI (0.946 ± 0.137). Our results indicated that the MDS-OAβ has a potential as a pre-screening tool for brain amyloidosis even in the MCI population.

PET scans are known to measure cumulative effects and capture topographic information of insoluble aggregates of cerebral Aβ [27, 28]. In contrast, blood-based biomarkers may reflect net rates of production and clearance of Aβ in near real time [5–7]. Thus, blood-based biomarkers and neuroimaging biomarkers may not necessarily manifest identical results and exhibit divergent timing in exhibiting abnormal findings. Taken this account, the 2023 Revised Criteria for Diagnosis and Staging of Alzheimer’s Disease has incorporated blood-based biomarkers as one of the two important pivots [29]. The criteria distinguished between imaging and fluid analyte biomarkers, and it suggested that the blood-based and neuroimaging biomarkers are not interchangeable but rather should be used complementary of each other. Moreover, even within the blood-based biomarkers of tau or T, the timing of abnormality onset also varied among. The p-tau 217, p-tau 181, and p-tau 231 became abnormal around the same time as A-PET [30–32], but MTBR-243 and non-phosphorylated tau fragments correlated more strongly with tau-PET [33, 34]. In this perspective, the MDS-OAβ might be used in tandem with other blood-based and neuroimaging biomarkers. Nevertheless, additional studies investigating association between MDS-OAβ and other blood-based and neuroimaging biomarkers are required to understand clinical utility of MDS-OAβ in the trajectory of AD.

It is not clear why patients with AD dementia showed lower MDS-OAβ level than patients with A-PET-positive MCI. The contemporary amyloid cascade hypothesis suggests that OAβ-dependent toxicity precedes amyloid plaque formation, and OAβ is present at earlier stages of the disease [35]. Others showed that the plasma ratio of Aβ_1–42_/Aβ_1–40_ increased in the early stages of AD but decreased as Aβ_1–42_, a monomer that is most prone to misfolding and aggregation, is deposited into Aβ plaques during progression of the disease [36–38]. Two longitudinal studies further showed that patients in the AD continuum had higher baseline level of Aβ_1–42_, and a significant decrement of plasma Aβ_1–42_ from baseline was associated with progression of MCI to AD dementia [39, 40]. Taken together, these findings indicate that MDS-OAβ decreased as more of the OAβ was aggregated into Aβ plaques along with advancement of AD severity. In line with our hypothesis, a study measuring CSF OAβ showed that OAβ increased at the onset of the disease, elevated as the disease progressed, and later fell as the disease became more severe [41]. However, longitudinal studies are needed to confirm our theory.

Our results confirmed those of previous studies that found a negative association between MDS-OAβ and cognitive functions [15]. More importantly, our findings that MDS-OAβ was positively associated with global and regional cerebral Aβ are also consistent with those of previous research [12]. Since the earlier studies only included NC and patients with AD dementia [12] or cerebral Aβ status-undefined subjects [15], they were not able to elucidate whether the association was due to cognitive function or cerebral Aβ deposition. We advanced previous research by including diverse patients along the AD continuum with cerebral Aβ status defined using A-PET and neurocognitive function measured using CERAD-K. Thus, we were able to show that MDS-OAβ was not only associated with neurocognitive measures but also with cerebral Aβ per se. Previous studies have indicated that the level of OAβ was correlated with the extent of synaptic loss, which would decrease hippocampal function [42]. Since we included patients from CN to AD dementia, OAβ associated synaptic loss and cognitive decline might have been more prominent. Nevertheless, multi-center studies using larger sample sizes are needed to verify our results.

In line with previous research, patients with A-PET negative MCI showed higher MDS-OAβ level than the NC [14]. Subthreshold level of Aβ deposition is known to increases risk of conversion to dementia in patients with A-PET negative MCI [43]. A significant number of A-PET-negative MCI subjects in our study might had subthreshold amyloid pathology, which might have contributed to higher oligomerization tendency than NC. In another perspective, MDS-OAβ is known to be associated with neurodegeneration [14]. Thus, a large proportion of subjects in the A-PET-negative MCI group might had higher neurodegeneration associated with non-amyloid pathology. Additional studies investigating correlation between MDS-OAβ and non-amyloid pathology are needed to confirm our speculation.

A study using VBM analysis already showed that MDS-OAβ was associated with cortical atrophy in cerebral Aβ status-undefined subjects, but the study mainly included cognitively normal older adults and those with AD dementia [14]. We advanced previous findings and found novel results that MDS-OAβ had a negative association with cortical thickness in subjects ranging from NC, A-PET-negative MCI, and A-PET-positive MCI. Recent studies suggested that soluble AβOs are associated with earlier stages of AD than the fibrillar Aβ of neuritic plaques [44]. Collectively, based on our group analysis which showed that MDS-OAβ was lower in AD dementia than in A-PET-positive MCI patients, MDS-OAβ could be closely linked with earlier neurodegenerative processes in the AD continuum. The detrimental downstream cascade of neurodegeneration after the disease has progressed to dementia could be more dependent on pathologies other than OAβ, such as tau proteins [45, 46]. The anatomical lesion, fusiform gyrus, showing negative association with MDS-OAβ, is also noteworthy. The fusiform gyrus is known to be involved in facial and lexical recognition, and it is one of the first brain areas to be affected during the progression of AD [47, 48]. In addition, a previous study demonstrated that atrophy of the fusiform gyrus occurs early in the AD trajectory as a consequence of Aβ within the hippocampus [49]. Others showed that fusiform gyrus is one of the regions exhibiting early elevation in tau-PET uptake [50]. A significant number of our participants might already had Aβ associated high cerebral tau burden and consequent neurodegeneration in fusiform gyrus. However, longitudinal studies combining multiple pathologies including Aβ, tau, and neurodegeneration are needed to elucidate neurobiological mechanisms underlying the role of OAβ in the AD continuum.

Our study contains several limitations. It was performed with samples collected from a single center, which may limit the generalizability of our results. This was a cross-sectional study, so the results can only elucidate correlations and have limited ability to interpret causal relations. We did not include patients with moderate to severe dementia or those with a CDR score of 2 or higher. Thus, we were unable to investigate whether MDS-OAβ decreases further as dementia severity progresses. We did not undertake tau-PET and plasma tau levels, so we were unable to investigate correlation between MDS-OAβ with that of phosphorylated or secreted AD tau and AD tau proteinopathy.

## Conclusions

We showed that MDS-OAβ increased when neurocognitive symptoms became clinically apparent, was heightened with higher cerebral Aβ burden, and decreased as the disease progressed further to dementia. MDS-OAβ was positively associated with cerebral Aβ burden throughout the different stages of AD. There also was a negative association between MDS-OAβ and cortical thickness among cognitively normal older adults and MCI patients. These findings suggest that the MDS-OAβ reflects earlier AD pathology, and it is not only associated with neurocognitive staging but is also correlated with the cerebral Aβ burden in patients along the AD continuum.

## Data Availability

The datasets used and/or analyzed during the current study are available from the corresponding author upon reasonable request.

## Abbreviations

3D-MPRAGE: Three-dimensional magnetization-prepared rapid gradient-echo
18F-FMM: 18F-flutemetamol
AD: Alzheimer’s disease
A-PET: Amyloid positron emission tomography
ADL: Activities of daily living
ANOVA: Analysis of variance
Aβ: β-Amyloid
Aβ40: β-Amyloid-40
Aβ42: β-Amyloid-42
BNT: Boston Naming Test
CABI: Catholic Aging Brain Imaging
CDR: Clinical dementia rating
CERAD-K: Consortium to Establish a Registry for Alzheimer’s Disease
CP: Constructional praxis
CR: Constructional recall
CSF: Cerebrospinal fluid
DSM: Diagnostic and Statistical Manual of Mental Disorders
EDTA: Ethylene-diamine-tetraacetic acid
FDG: Fluorodeoxyglucose
FWHM: Full width at half-maximum
GM: Gray matter
MCI: Mild cognitive impairment
MDS-OAβ: Multimer detection system-oligomeric Aβ
NC: Normal control
NINCDS-ADRDA: National Institute of Neurological and Communicative Disorders and Stroke and AD and Related Disorders Association
OAβ: Oligomeric forms of Aβ
p-Tau: Phosphorylated tau
PC: Precuneus
PCC: Posterior cingulate cortex
PiB: Pittsburgh compound B
SUVR: Standardized uptake value ratio
t-Tau: Total tau
VBM: Voxel-based morphometry
VF: Verbal fluency
WLM: Word list memory
WLR: Word list recall
WLRc: Word list recognition
WM: White matter

## References

[1] Alzheimer's disease facts and figures (2022). Alzheimers Dement.

[2] Jack CR, Bennett DA, Blennow K, Carrillo MC, Dunn B, Haeberlein SB (2018). NIA-AA Research Framework: Toward a biological definition of Alzheimer's disease. Alzheimers Dement.

[3] Rabinovici GD (2021). Controversy and Progress in Alzheimer's Disease - FDA Approval of Aducanumab. N Engl J Med.

[4] Larkin HD (2023). Lecanemab Gains FDA Approval for Early Alzheimer Disease. JAMA.

[5] Janelidze S, Teunissen CE, Zetterberg H, Allue JA, Sarasa L, Eichenlaub U (2021). Head-to-Head Comparison of 8 Plasma Amyloid-beta 42/40 Assays in Alzheimer Disease. JAMA Neurol.

[6] Janelidze S, Bali D, Ashton NJ, Barthelemy NR, Vanbrabant J, Stoops E (2023). Head-to-head comparison of 10 plasma phospho-tau assays in prodromal Alzheimer's disease. Brain.

[7] Karikari TK, Pascoal TA, Ashton NJ, Janelidze S, Benedet AL, Rodriguez JL (2020). Blood phosphorylated tau 181 as a biomarker for Alzheimer's disease: a diagnostic performance and prediction modelling study using data from four prospective cohorts. Lancet Neurol.

[8] Barthelemy NR, Horie K, Sato C, Bateman RJ. Blood plasma phosphorylated-tau isoforms track CNS change in Alzheimer's disease. J Exp Med. 2020;217(11):e20200861. 10.1084/jem.20200861 PMC7596823 32725127

[9] An SSA, Lee BS, Yu JS, Lim K, Kim GJ, Lee R (2017). Dynamic changes of oligomeric amyloid beta levels in plasma induced by spiked synthetic Abeta42. Alzheimers Res Ther.

[10] An SS, Lim KT, Oh HJ, Lee BS, Zukic E, Ju YR (2010). Differentiating blood samples from scrapie infected and non-infected hamsters by detecting disease-associated prion proteins using Multimer Detection System. Biochem Biophys Res Commun.

[11] Lim K, Kim SY, Lee B, Segarra C, Kang S, Ju Y (2015). Magnetic microparticle-based multimer detection system for the detection of prion oligomers in sheep. Int J Nanomedicine.

[12] Wang MJ, Yi S, Han JY, Park SY, Jang JW, Chun IK (2017). Oligomeric forms of amyloid-beta protein in plasma as a potential blood-based biomarker for Alzheimer's disease. Alzheimers Res Ther.

[13] Meng X, Li T, Wang X, Lv X, Sun Z, Zhang J (2019). Association between increased levels of amyloid-beta oligomers in plasma and episodic memory loss in Alzheimer's disease. Alzheimers Res Ther.

[14] Youn YC, Kang S, Suh J, Park YH, Kang MJ, Pyun JM (2019). Blood amyloid-beta oligomerization associated with neurodegeneration of Alzheimer's disease. Alzheimers Res Ther.

[15] Lee JJ, Choi Y, Chung S, Yoon DH, Choi SH, Kang SM, et al. Association of Plasma Oligomerized Beta Amyloid with Neurocognitive Battery Using Korean Version of Consortium to Establish a Registry for Alzheimer's Disease in Health Screening Population. Diagnostics (Basel). 2020;10(4):237. 10.3390/diagnostics10040237 PMC7236003 32326061

[16] Youn YC, Lee BS, Kim GJ, Ryu JS, Lim K, Lee R (2020). Blood Amyloid-beta Oligomerization as a Biomarker of Alzheimer's Disease: A Blinded Validation Study. J Alzheimers Dis.

[17] Hutton C, Draganski B, Ashburner J, Weiskopf N (2009). A comparison between voxel-based cortical thickness and voxel-based morphometry in normal aging. Neuroimage.

[18] Lee JH, Lee KU, Lee DY, Kim KW, Jhoo JH, Kim JH (2002). Development of the Korean version of the Consortium to Establish a Registry for Alzheimer's Disease Assessment Packet (CERAD-K): clinical and neuropsychological assessment batteries. J Gerontol B Psychol Sci Soc Sci.

[19] Dubois B, Feldman HH, Jacova C, Dekosky ST, Barberger-Gateau P, Cummings J (2007). Research criteria for the diagnosis of Alzheimer's disease: revising the NINCDS-ADRDA criteria. Lancet Neurol.

[20] Tay L, Lim WS, Chan M, Ali N, Mahanum S, Chew P (2015). New DSM-V neurocognitive disorders criteria and their impact on diagnostic classifications of mild cognitive impairment and dementia in a memory clinic setting. Am J Geriatr Psychiatry.

[21] Babapour Mofrad R, Scheltens P, Kim S, Kang S, Youn YC, An SSA (2021). Plasma amyloid-beta oligomerization assay as a pre-screening test for amyloid status. Alzheimers Res Ther.

[22] Cardinale F, Chinnici G, Bramerio M, Mai R, Sartori I, Cossu M (2014). Validation of FreeSurfer-estimated brain cortical thickness: comparison with histologic measurements. Neuroinformatics.

[23] Lim HK, Jung WS, Ahn KJ, Won WY, Hahn C, Lee SY (2012). Regional cortical thickness and subcortical volume changes are associated with cognitive impairments in the drug-naive patients with late-onset depression. Neuropsychopharmacology.

[24] Fischl B, Dale AM (2000). Measuring the thickness of the human cerebral cortex from magnetic resonance images. Proc Natl Acad Sci U S A.

[25] Thurfjell L, Lilja J, Lundqvist R, Buckley C, Smith A, Vandenberghe R (2014). Automated quantification of 18F-flutemetamol PET activity for categorizing scans as negative or positive for brain amyloid: concordance with visual image reads. J Nucl Med.

[26] https://www.jamovi.org TjpjVCSRf.

[27] Lowe VJ, Lundt ES, Albertson SM, Przybelski SA, Senjem ML, Parisi JE (2019). Neuroimaging correlates with neuropathologic schemes in neurodegenerative disease. Alzheimers Dement.

[28] Fleisher AS, Pontecorvo MJ, Devous MD, Lu M, Arora AK, Truocchio SP (2020). Positron Emission Tomography Imaging With [18F]flortaucipir and Postmortem Assessment of Alzheimer Disease Neuropathologic Changes. JAMA Neurol.

[29] Jeffrey Scott Andrews TGB, Teresa Buracchio, Maria C. Carrillo, Billy Dunn, Ana Graf, Oskar Hansson, Carole Ho, Clifford R. Jack Jr., William Jagust, Eliezer Masliah, Eric McDade, José Luis Molinuevo, Ozioma Okonkwo, Luca Pani, Michael Rafii, Laurie Ryan, Phillip Scheltens, Eric Siemers, Heather Snyder, Reisa Sperling, Charlotte E. Teunissen. Revised Criteria for Diagnosis and Staging of Alzheimer's Disease: Alzheimer’s Association Workgroup. Alzheimer's Association International Conference 20232023.

[30] Barthelemy NR, Li Y, Joseph-Mathurin N, Gordon BA, Hassenstab J, Benzinger TLS (2020). A soluble phosphorylated tau signature links tau, amyloid and the evolution of stages of dominantly inherited Alzheimer's disease. Nat Med.

[31] Janelidze S, Berron D, Smith R, Strandberg O, Proctor NK, Dage JL (2021). Associations of Plasma Phospho-Tau217 Levels With Tau Positron Emission Tomography in Early Alzheimer Disease. JAMA Neurol.

[32] Mattsson-Carlgren N, Andersson E, Janelidze S, Ossenkoppele R, Insel P, Strandberg O (2020). Abeta deposition is associated with increases in soluble and phosphorylated tau that precede a positive Tau PET in Alzheimer's disease. Sci Adv.

[33] Horie K, Barthelemy NR, Sato C, Bateman RJ (2021). CSF tau microtubule binding region identifies tau tangle and clinical stages of Alzheimer's disease. Brain.

[34] Montoliu-Gaya L, Benedet AL, Tissot C, Vrillon A, Ashton NJ, Brum WS (2023). Mass spectrometric simultaneous quantification of tau species in plasma shows differential associations with amyloid and tau pathologies. Nat Aging.

[35] Zott B, Simon MM, Hong W, Unger F, Chen-Engerer HJ, Frosch MP (2019). A vicious cycle of beta amyloid-dependent neuronal hyperactivation. Science.

[36] Cohen SI, Linse S, Luheshi LM, Hellstrand E, White DA, Rajah L (2013). Proliferation of amyloid-beta42 aggregates occurs through a secondary nucleation mechanism. Proc Natl Acad Sci U S A.

[37] Economou NJ, Giammona MJ, Do TD, Zheng X, Teplow DB, Buratto SK (2016). Amyloid beta-Protein Assembly and Alzheimer's Disease: Dodecamers of Abeta42, but Not of Abeta40, Seed Fibril Formation. J Am Chem Soc.

[38] van Oijen M, Hofman A, Soares HD, Koudstaal PJ, Breteler MM (2006). Plasma Abeta(1–40) and Abeta(1–42) and the risk of dementia: a prospective case-cohort study. Lancet Neurol.

[39] Mayeux R, Honig LS, Tang MX, Manly J, Stern Y, Schupf N (2003). Plasma A[beta]40 and A[beta]42 and Alzheimer's disease: relation to age, mortality, and risk. Neurology.

[40] Gabelle A, Richard F, Gutierrez LA, Schraen S, Delva F, Rouaud O (2013). Plasma amyloid-beta levels and prognosis in incident dementia cases of the 3-City Study. J Alzheimers Dis.

[41] Holtta M, Hansson O, Andreasson U, Hertze J, Minthon L, Nagga K (2013). Evaluating amyloid-beta oligomers in cerebrospinal fluid as a biomarker for Alzheimer's disease. PLoS ONE.

[42] Lacor PN, Buniel MC, Chang L, Fernandez SJ, Gong Y, Viola KL (2004). Synaptic targeting by Alzheimer's-related amyloid beta oligomers. J Neurosci.

[43] Kim HJ, Oh JS, Lim JS, Lee S, Jo S, Chung EN (2022). The impact of subthreshold levels of amyloid deposition on conversion to dementia in patients with amyloid-negative amnestic mild cognitive impairment. Alzheimers Res Ther.

[44] Shankar GM, Li S, Mehta TH, Garcia-Munoz A, Shepardson NE, Smith I (2008). Amyloid-beta protein dimers isolated directly from Alzheimer's brains impair synaptic plasticity and memory. Nat Med.

[45] Bejanin A, Schonhaut DR, La Joie R, Kramer JH, Baker SL, Sosa N (2017). Tau pathology and neurodegeneration contribute to cognitive impairment in Alzheimer's disease. Brain.

[46] Tanner JA, Iaccarino L, Edwards L, Asken BM, Gorno-Tempini ML, Kramer JH (2022). Amyloid, tau and metabolic PET correlates of cognition in early and late-onset Alzheimer's disease. Brain.

[47] Ma D, Fetahu IS, Wang M, Fang R, Li J, Liu H (2020). The fusiform gyrus exhibits an epigenetic signature for Alzheimer's disease. Clin Epigenetics.

[48] Koychev I, Hofer M, Friedman N (2020). Correlation of Alzheimer Disease Neuropathologic Staging with Amyloid and Tau Scintigraphic Imaging Biomarkers. J Nucl Med.

[49] Chang YT, Huang CW, Chen NC, Lin KJ, Huang SH, Chang WN (2016). Hippocampal Amyloid Burden with Downstream Fusiform Gyrus Atrophy Correlate with Face Matching Task Scores in Early Stage Alzheimer's Disease. Front Aging Neurosci.

[50] Insel PS, Mormino EC, Aisen PS, Thompson WK, Donohue MC (2020). Neuroanatomical spread of amyloid beta and tau in Alzheimer's disease: implications for primary preventio. Brain Commun.

